# The Physiology of the Circulation

**Published:** 1860-06

**Authors:** John C. Dalton

**Affiliations:** Professor of Physiology and Microscopic Anatomy


*The Physiology of the Circulation. A Course of Lectures delivered at the College of Physicians and Surgeons, New York, in the Fall Term of* 1859. By JOHN C. DALTON, JR., M.D., Professor of Physiology and Microscopic Anatomy.

### LECTURE VI.

(SEPTEMBER 28.)

Venous Circulation—Structure of the Veins—Their Inosculation—Pressure of Blood in the Veins—Fluctuating Character of Venous Current—Influence of Muscular Contraction—Of Respiratory Movements—Pulsation of Veins near the Thorax—Force of Aspiration exerted by the Chest—Its Effect on the Movement of the Blood—Experiment—Introduction of Air into the Veins—Mode in which it produces Death—Experiment—Introduction of Air in Surgical Operations—Cases—Symptoms—Instances of Recovery—Post-mortem Appearances in Fatal Cases—Means of Diagnosis—Introduction of Air by Vessels of Uterus—Recorded Cases—Case of Dr. Swinburne.

To-day, gentlemen, we shall pass from the consideration of the arterial to that of the venous system; from the study of the circulation of the blood in the arteries, to its course through the veins, in its returning passage from the capillaries to the heart.

You already know the general structure and arrangement of the veins, and the main peculiarities which distinguish them from the arterial tubes. I shall not, therefore, take up your time by dwelling upon these points, but shall ask your attention more particularly to the conditions which affect the movement of the blood in these vessels, and the variations of the venous circulation in different parts of the body.

Although the veins are provided with walls which are very much thinner and less elastic than those of the arteries, yet, contrary to what we might expect, their capacity for *resistance to pressure* is equal, or even superior, to that of the arterial tubes. Milne Edwards has collected the results of various experiments, which show that the veins will sometimes resist a pressure which is sufficient to rupture the walls of the arteries. In one instance the jugular vein supported, without breaking, a pressure equal to a column of water 148 feet in height; and in another, the iliac vein of a sheep resisted a pressure of more than four atmospheres. The portal vein was found capable of resisting a pressure of six atmospheres; and in one case, in which the aorta of a sheep was ruptured by a pressure of 158 pounds, the vena cava of the same animal supported a pressure equal to 176 pounds.

This resistance of the veins is to be attributed to the large proportion of white fibrous tissue which enters into their composition; the same tissue which forms nearly the whole of the tendons and fasciæ, and which is distinguished by its density and unyielding nature.

The *elasticity* of the veins, however, is much less than that of the arteries. When they are filled with blood, they enlarge to a certain size, and collapse again when the pressure is taken off; but they do not react by virtue of an elastic resilience, or, at least, only to a slight extent, as compared with the arteries. Accordingly, when the arteries are cut across, as we know, and emptied of blood, they still remain open and pervious, retaining the tubular form, on account of the elasticity of their walls; while, if the veins be treated in the same way, their sides simply fall together and remain in contact with each other.

Another peculiarity of the venous system is the *abundance of the separate channels*, which it affords, for the flow of blood from the periphery towards the centre. The arteries, as you know, pass directly from the heart outward, each separate branch, as a general rule, going to a separate region, and supplying that part of the body with all the blood which it requires; so that the arterial system is kept constantly filled to its entire capacity with the blood which passes through it.

That, however, is not the case with the veins. In this injected preparation of the vascular system, you observe, we have two, three, four, or even five veins, coming together from the same region of the body. You will notice, also, that there are abundant inosculations between the different veins. The deep veins which accompany the brachial artery inosculate freely with each other, and also with the superficial veins of the arm. In the veins coming from the head, we have the external jugular communicating with the thyroid veins, the anterior jugular, and the brachial veins. The external and internal jugulars communicate with each other, and the two thyroid veins also form an abundant plexus in front of the trachea.

Thus the blood, coming from the extremities towards the heart, flows, not in a single channel, but in many channels; and as these channels communicate freely with each other, the blood passes sometimes through one of them, and sometimes through another.

We find, accordingly, that the venous system, as a whole, is not, in its normal condition, constantly distended with blood. One part of it, on the contrary, may be full, while another part is comparatively empty. One vein may be compressed, while another is left free; and a fluctuation of the blood, therefore, may readily take place from one portion of the venous system to another, as different parts are alternately filled or emptied.

On account of this arrangement, you will see at once that the entire pressure of the blood in the veins cannot be judged of in precisely the same manner as that in the arteries. We found yesterday, in studying the pressure of the blood in the arteries, that this pressure was the same, or nearly the same, throughout the whole arterial system. It is the same in a large artery as in a small one; and the difference is not great, whether we apply the mercurial guage to an artery near the heart or to one at a distance from it. If we apply the guage to the aorta, we get the pressure of all the blood passing through this tube to the whole body; and the pressure indicated must be sufficient to distribute that quantity of blood to the entire system. If we apply the guage, on the other hand, to the femoral artery, we there find a quantity of blood, which is adequate for the circulation of the lower extremity, propelled by a corresponding force. These relations remaining the same throughout the entire system, the indications of the guage are not essentially different, whether we apply it to one artery or to another.

But it is not so with the venous system. In this part of the circulatory apparatus, the pressure diminishes, very distinctly, from the periphery toward the centre. It has been found that if the mercurial guage be applied, at the same time, to the carotid artery, the metatarsal vein, and the jugular vein, the column of mercury may indicate a pressure of 165 millimetres for the carotid artery, but only 27 millimetres for the metatarsal vein, and 9 millimetres for the jugular. Volkmann experimented in a similar manner upon a horse, and found that the pressure in the small veins of the neck was 44 millimetres, while that in the jugular was only 21½ millimetres. In a goat, the pressure in the facial vein was found to be 41 millimetres, and that in the jugular vein only 18. So that the cardiometer indicates a diminution of pressure from the arteries to the veins, and a further diminution, in the veins themselves, from the periphery toward the heart.

This is not, however, because the force, with which the blood is returned to the heart by the veins, is really less than that by which it is distributed outward by the arteries, but because its passage through the venous system takes place by so many collateral channels, which communicate with each other by inosculating branches. If we apply the mercurial guage, for example, to one of the veins of the extremities, it does not indicate the entire pressure of the blood, as it returns towards the heart; for a large portion flows off by other veins, and so finds its way back, by a different course, to the centre of the circulation.

A very ingenious experiment, by Poisseuille, has shown that this difference of pressure in the venous system is more apparent than real; and that it is owing to the cause which I have just stated. If the cardiometer be placed, at the same time, upon the artery of a limb and upon its accompanying vein, we shall find, as I have said, that the pressure in the vein is much less than that in the artery. But if all the rest of the limb be surrounded with a ligature, so as to compel the whole of the blood to return by a single vein, the pressure in the vein will then be seen to rise, until it is nearly or quite equal to that in the corresponding artery. Undoubtedly, there are some organs in the body where the venous pressure would be even greater than the arterial, owing to an increase in the mass of the blood as it passes through the capillary circulation. But these variations I shall speak of more particularly hereafter.

The blood returns to the heart, therefore, by a considerable number of inosculating venous channels; and the pressure of the blood in the veins diminishes, for that reason, as we approach from the circumference to the centre.

There is another peculiarity of the venous circulation, still more marked than that which I have just described. It relates to the *physical impulses* under which the blood moves through the venous system.

In the veins, the blood does not flow with a regular rhythmical movement, like that of the arteries. In them, there is no regularly returning expansion and collapse, corresponding with the pulsations of the heart. The blood flows through the veins with an intermittent motion, it is true, but this movement is irregular, and not rhythmical. It is to be seen, more particularly, in the veins of the external parts of the trunk, in the neck and extremities; in all those parts, indeed, where the venous channels pass between muscles, or through the muscular tissue. The voluntary muscles, in these parts, are continually contracting and relaxing. Their contraction compresses the veins which pass between them, and thus drives the venous blood onward, in its passage toward the heart.

This movement is provided for, as you know, by the valves of the veins, which are so arranged that they open toward the heart, and shut backward toward the capillaries.

Here you have the jugular vein of a dog, which I removed this morning, cut open in such a way as to show the valves. They are arranged, as you observe, in pairs, upon the internal surface of the vessel. They have the form of membranous festoons; and by directing a stream of water or of air against them, in a backward direction, they may be made to fill out and become distended, with their convexities looking toward the cavity of the vein.

Here is the jugular vein of an ox, still unopened; by tying one end of it, and then inflating it with air, the position of the valves in the interior may be readily distinguished by the bulging prominences which show themselves at various points. These valves are mostly double, but there is one situated near the end of the vein which is composed of three distinct festoons. When distended, their edges come in contact with each other, like those of the pulmonary or aortic valves.

These valves prevent the regurgitation of the blood when the veins are subjected to external compression.

You will see, then, how it is that the venous circulation is influenced by the action of the voluntary muscles. This action is irregular, but nevertheless is constantly recurring, at nearly all periods of the day. The muscles contract, and drive the blood onward toward the heart. They relax, and the veins fill up again from behind. The blood is moved through the veins, not by a contraction of the walls of the veins themselves, but by that of the voluntary muscles situated outside of them. The action of the voluntary muscles, therefore, in the venous system, replaces, to a considerable extent, that of the heart in the arterial system.

The circulation in the veins, accordingly, differs from that in the arteries by the irregularity of its movement. The flow of blood in the arterial system is regular and rhythmical; in the venous system it is variable and fluctuating. The arteries are constantly full of blood; and the arterial pressure is constantly the same, or is varied only by the pulsations of the heart. The veins, on the other hand, are alternately filled and emptied by the muscular contractions, and the pressure varies in each vein, as its collateral branches are open or obstructed.

There is another peculiarity still, in the venous circulation, which I will not omit to mention. It is that the venous current is influenced, to a great degree, by the movements of the chest. If we examine any large vein in the immediate neighborhood of the thorax, we shall find that it alters very perceptibly in appearance with the movements of respiration. At every movement of expiration the vein becomes turgid, and at every inspiration it collapses again to its former size. Now, what are these movements of respiration, and why should they affect the flow of blood through the veins? We have already examined them to some extent, in connection with their effect on the arterial pressure. In the movement of inspiration, the air is drawn into the interior of the thorax by a lifting action of the ribs and a descent of the diaphragm. This is, in reality, a *suction* movement, and, when it takes place, all the fluids are drawn into the chest which can gain access to its cavity—the air through the trachea, and the blood through the veins.

At each inspiration, therefore, the veins in the neighborhood of the chest collapse, and at every expiration become distended. This alternate movement of expansion and collapse is very marked whenever the respiration becomes rapid or laborious.

Now, you will recollect that this effect of the respiratory movements was very perceptible yesterday, when the mercurial gauge was applied to one of the arteries. We saw then that the pressure of the blood in the vessel was influenced by three different causes: *first*, the constant and steady reaction of the arterial system; *second*, the contractions of the heart, which produced an oscillation of five to fifteen millimetres; and *thirdly*, there was another oscillation, which was synchronous with the movements of the chest. At every inspiration the column of mercury fell, and at every expiration it rose to a higher level.

A somewhat similar experiment has been tried with the veins. A bent glass tube was introduced, by Barry, into one of the large veins near the heart, and secured by ligature. The other end of the tube dipped into a vessel containing a colored fluid, and at every inspiration the colored fluid was seen to rise in the tube, while at the moment of expiration it descended to its former level.

We find, therefore, that whenever the chest expands, the blood flows into it from the neighboring veins, in the same way as air flows in through the trachea; and when inspiration ceases and expiration begins, the veins fill again up from behind, and thus regain their original size. The force by which the blood is thus drawn toward the chest, by the respiratory movements, is called the force of *aspiration.*

In order to exhibit this action of the veins, I will now etherize the animal which we have here, (a cat,) and afterward expose the external jugular, as it passes down the neck. You now see the vein, running from above downward, over the anterior surface of the sterno-mastoid muscle. It is moderately distended with blood, but we do not see, as yet, any changes in its size, corresponding with the movements of respiration. There is no emptying of the vein at the time of inspiration, nor any distention at the time of expiration. This is because the portion of the jugular which we are now examining is at too great a distance from the chest, to show the effect of the thoracic movements.

If, however, we uncover a portion of the vein which is nearer the entrance of the thorax, it will then, no doubt, be visibly affected by the movements of respiration. I now expose the vein farther down, dissecting away the muscles which cover it, nearly to the point where it enters the cavity of the chest. You see now that at the lower extremity of the wound, the vein becomes partially collapsed and emptied at each movement of inspiration; whereas at the upper part of the wound, where the vein passes over the sterno-mastoid muscle, no such movement is visible.

You can see, in fact, at the lower part of the vein two different movements at the same time. One is the alternate expansion and collapse of the vein, synchronous with the rise and fall of the chest in respiration. The other is a more rapid, tremulous motion, corresponding with the pulsations of the heart, the impulse of which is communicated to this portion of the vein.

Now, with regard to this force of aspiration exerted by the chest upon the venous blood, there are two circumstances which it is important to remember.

In the first place, in the ordinary condition of the respiratory movements, when the breathing is perfectly calm and regular, there is but very little visible effect produced upon the veins, even in the immediate neighborhood of the chest. The collapse of the veins at the moment of inspiration is hardly perceptible to the eye. I do not doubt that an effect is really produced upon the venous circulation even then, and that the flow of blood toward the heart is favored by the movements of the chest. But at this time, the force of aspiration is so gentle and uniform that the veins fill up from behind by the venous current as fast as they are emptied in front by the expansion of the chest. If you etherize an animal, therefore, as we have done in this instance, and then expose the jugular vein at the lower part of the neck, so long as the respiration is quiescent you will not see any marked fluctuation in the vein externally. It is only when the movements of respiration become hurried or laborious that this fluctuation becomes manifest. For then, the vein is emptied by the forcible expansion of the chest faster than it can fill up from behind, and of course it collapses visibly at each inspiration. As the breathing becomes more violent, the alternate movement of collapse and distention is more decided, and corresponds in intensity with the movements of respiration.

For the same reason, the venous fluctuation is most marked in continued struggling or crying. The breathing is then performed by long and continuous expirations, alternating with quick, short, and violent inspirations. Accordingly, the veins suffer a corresponding distention and collapse at that time, when these movements would be hardly perceptible in the ordinary condition of the respiration.

In the second place, when the breathing is thus hurried and laborious, the force of aspiration only acts upon the veins *in the immediate neighborhood of the chest.* You saw, in the experiment which we have just tried, that while the venous fluctuation was quite visible in the lower part of the jugular, it was not at all so in the upper part of the same vein. Why is it, that this emptying and filling of the veins should take place at the upper part of the chest, and not in the middle of the neck? The explanation of this fact is very important in connection with the movement of the blood, and also in regard to a peculiar surgical accident which I shall speak of in a few moments. Why is it, then, that the venous fluctuation should be visible at this time in the immediate neighborhood of the thorax, and not at a distance from it?

It is simply because the walls of the veins are loose, flexible, and liable to collapse. They cannot maintain their tubular form, when suddenly emptied by the force of suction; and, therefore, when the veins are affected by the movements of the chest, not only is the blood which they contain drawn into the thorax, but their walls are at the same time drawn together, and the veins collapse.

We find, accordingly, that the emptying of the veins, by a violent inspiration, is not felt at a distance from the spot where the force is originally exerted.

We can see the same thing perfectly well, by experimenting upon any flexible tube with yielding walls. Here, for example, is a syringe, to the nozzle of which I have attached a flexible india-rubber tube with thin walls. I dip the lower extremity of the india-rubber tube into a vessel of water. Now, if I withdraw the piston of the syringe slowly and gently, the water is drawn up into the tube, as the blood is drawn through the veins by a quiet and easy inspiration. But if I withdraw the piston by a sudden and forcible motion, the walls of the tube, you observe, immediately collapse, and no water can gain admittance into the syringe.

This is precisely what happens with the veins, in regard to the movements of the thorax. They collapse so readily, in a forcible inspiration, that the only blood drawn into the chest is that which was to be found at a very short distance from its cavity. It makes a great difference, therefore, in the movement of the blood, whether the motions of respiration are performed quietly or forcibly. So long as the respiration is quiet, each movement of inspiration draws the blood, by a gentle motion, from the whole venous system toward the chest; but as soon as the breathing becomes violent, the forcible expansion of the chest causes a collapse of the veins in its neighborhood, and no longer assists in the circulation of the blood.

There are various accidents, gentlemen, which are liable to happen during surgical operations, in consequence of injury done to the veins. One of these, of course, is hæmorrhage; but this, as a general thing, is the least important. In nearly all cases, venous hæmorrhage is easily controlled, except where a large number of veins have been wounded simultaneously. Compression or ligature is usually quite sufficient to arrest the bleeding. At one time, a great deal of dread was entertained by surgeons with regard to the ligature of veins; it being considered as hazardous, owing to the supposed irritability of these vessels, and their liability to become inflamed after the application of the ligature.

So far as I am aware, this feeling has very much diminished of late years. In regard to my own experience, I have found, as a general thing, in the lower animals, little or no difference in irritability between the arteries and veins. I tie the veins, in an operation, with as little hesitation as I would place the ligature upon an artery, and with no more anticipation of an unfavorable result. And I do not know that, in these animals, I have ever seen unpleasant consequences ensue.

A more dangerous, and still more dreaded, occurrence, which may sometimes happen in operating upon the veins, is the *introduction of air* into the venous system. This accident has received, from time to time, a great deal of attention from practical surgeons. In the early annals of surgery, cases of sudden death during operations were sometimes recorded, the reasons for which were not then understood, but which were afterward supposed to be owing to this cause.

There are one or two remarks which I wish to make at the outset, in regard to this introduction of air into the veins. The accident is not, of itself, so constantly and excessively dangerous as we might be led to suppose. It is often thought that in a surgical operation the slightest admission of air into the veins must be instantly and inevitably fatal; that the smallest quantity of aëriform fluid in the venous system is enough to paralyze the heart or the brain, and put a stop to all the vital operations. This, however, is certainly not the case. We may sometimes introduce a considerable quantity of air into the blood, without producing a fatal, or even an immediately serious effect.

The effect which *is* produced depends, to a very great degree, not upon the simple fact that air is introduced, but upon the quantity in which it is introduced, and upon the manner of its introduction. It depends even still more upon the manner of its introduction than upon its quantity. A very small amount may be thrown into the veins of an animal at almost any time, without producing death; and even a large quantity, if thrown in slowly and gradually, may be almost equally harmless. It is only when a considerable quantity is introduced suddenly, that unpleasant consequences follow, and a fatal result almost necessarily produced.

Now, why is it that such a difference should exist in the effects of the same operation, done in two different ways? We shall see the reason for this difference, when we understand the mechanism by which air in the veins acts in producing death.

It is by its mechanical effect, and not by any chemical or poisonous agency, that air, when mixed with the blood, causes death. It is air *in an aëriform, and not in a liquid condition*, that blocks the circulation and paralyzes the heart. The gases of the atmosphere have no injurious property in themselves. In fact, both oxygen and nitrogen, as you already know, are normal ingredients of the blood, and are naturally held in solution in the circulating fluid. Accordingly, if air be introduced into the veins in small quantity, it is immediately dissolved by the blood, and, in that condition, does no harm. If even a large quantity be injected, so slowly that the blood can dissolve it as fast as it is introduced, it produces no bad effect, and passes without injury through the circulation.

But if the air be thrown in abundantly and rapidly, it fails to be dissolved. It then remains mixed with the blood in the form of gaseous bubbles, and interferes with the circulation to such a degree that death is very soon the result.

Now we can very easily see, by exposing one of the veins in this animal, (a cat,) and injecting air, that a small quantity does not immediately or necessarily produce death. I have already uncovered the femoral vein, as it passes up the inside of the thigh, alongside the femoral artery. I have here, also, a syringe which is perfectly air-tight; for you see, on dipping the nozzle into a vessel of water, that the slightest pressure on the piston produces an abundant escape of air-bubbles. We can, therefore, use the instrument for the purpose of injecting air into the vein.

I now make an opening in the vein, introduce into it the nozzle of the syringe, and slowly depress the piston to a certain extent. Thus far, you observe, no immediate effect has been produced on the circulation, and yet air in considerable quantity has been introduced into the circulation, for I can hear very distinctly, on listening at the chest, a *churning* sound, which indicates that air is already mixed with the blood in the right cavities of the heart. This churning sound is a very curious phenomenon, and is almost always to be heard when air has been thrown into the veins in considerable quantity. For the air, immediately after its introduction, is carried directly to the right side of the heart, and is there whipped into a minute foam, by the motion of the tendinous cords and valves of the heart. This gives rise to a characteristic sound, like a moist râle, or double souffle, which may be heard through the walls of the chest.

In this instance, the air has been introduced so gently that the quantity which is now in the heart can undoubtedly be disposed of by solution, and therefore will not cause any serious injury. I no longer hear the churning sound of the air in the cardiac cavities, and I presume, therefore, that it has already been nearly or quite dissolved in the blood.

I will now, by means of the same syringe, force into the vein a larger quantity of air, and by a sudden motion; which, I presume, will prove fatal to the animal. I now hear again the churning sound of air in the right ventricle. The heart labors much more than at first, though, you observe, the movements of respiration continue, and the cardiac pulsations are still regular. Now, however, they are diminishing in force, and the movements of respiration, also, are already nearly at an end. You see, therefore, that the rapid introduction of air has proved fatal within a few seconds; while a smaller quantity may be introduced gradually, without any serious effect.

Now let us see what is the mechanism of this accident. How is it that air produces death, when mixed with the blood in an undissolved condition?

The air, when thrown into the veins, as we have seen, is immediately carried to the right side of the heart. It thence passes out by the pulmonary artery and its branches, to the capillaries of the lungs, and fills these vessels with finely-divided aëriform bubbles. But the capillaries present an obstacle to its further passage. The air clings to the internal surface of the minute blood-vessels, and becomes entangled in the capillary plexus. It can pass easily enough through the arteries, and through the veins; but it cannot readily find its way through the capillaries. The vessels of the lungs thus become blocked and obstructed, the blood is cut off from the left side of the heart and the arterial system, and in this way the circulation suddenly comes to an end.

It makes a great difference, therefore, whether we inject the air into an artery or into a vein. If we throw it into an artery, it is arrested in the capillaries of that region of the body alone, and only a part of the circulation becomes obstructed. But if we throw it into a vein, it is at once conducted to the heart, thence carried to the lungs, and the whole vascular system is then deranged, by the stoppage of the pulmonary circulation.

Now, in order to ascertain whether this be really the case, I will make an autopsy of the animal that we have just killed, and see what has become of the air which was injected into the veins. On opening the chest, you see, gentlemen, that the heart still pulsates feebly. The air contained in the right ventricle is distinctly visible through the pericardium, which is still unopened. You observe, also, some bubbles of air in the superior vena cava, into which it has passed by regurgitation from below.

You see, furthermore, that the pericardium is very much distended by the heart. The heart itself is so distended, and fills so completely the pericardial cavity, that it is difficult to pinch up the fibrous membrane with the forceps, and separate it from the surface of the heart. Now, however, I can succeed in cutting open the pericardium, and you see that the heart immediately protrudes through the opening, by a kind of hernia. Everything indicates an unnatural distention of the cardiac cavities.

This distention is owing to the presence of air. On striking the heart with the handle of a scalpel, you observe it returns a tympanitic sound, very similar to that of the stomach, and altogether different from the dull, solid sound of a heart filled with blood. We have, then, air in the superior and inferior vena cava, air in the right auricle, and air in the right ventricle. No doubt, there is air also in the pulmonary artery and its branches. But, in all probability, this air has not passed through the pulmonary circulation, and is not to be found in the left cavities of the heart.

In order to make this certain, however, I will first open the left cavities, and afterward the right.

I now open the left auricle and ventricle; and what escapes? Only a few drops of bright scarlet blood! Here is already, then, a remarkable difference in the appearance of the left cavities from what we saw the other day in the animal killed by stoppage of the respiration. In that case, both the left auricle and the left ventricle were distended, at the moment of death, with dark venous blood. Here, on the contrary, the blood has not been thrown backward upon the heart from the capillaries, but has been stopped in its passage through the lungs. Accordingly, there is but little blood in the left ventricle, and that little is of the natural arterial color.

You see, furthermore, that there are no bubbles mixed with the blood in the left cavities of the heart. The air, therefore, has not passed through the pulmonary circulation.

I will now open the right auricle and ventricle. Immediately there escapes a large quantity of bright frothy blood, formed of minute bubbles of air intimately mixed with the blood, and beaten up into a foam by the action of the heart. It is this foam which obstructed the capillaries of the lungs, and put an end to the pulmonary circulation.

Now, these are the appearances usually found on opening an animal, killed by injection of air into the veins. Air in the vena cava, air in the right auricle, the right ventricle, and the pulmonary artery; but none in the pulmonary veins, nor in the left cavities of the heart. The ordinary pressure of the circulation is not sufficient to force the air through the blood-vessels of the lungs. Still, this may be effected by a greater amount of pressure. If we inject the air forcibly into the veins, under a steady and continued pressure for thirty or forty seconds, we may overcome the resistance of the capillaries, and compel some of the air to pass through them. Under these circumstances, air-bubbles may be also found, in small quantity, in the left side of the heart.

I have myself succeeded, in this way, in forcing air through the capillaries of the intestine, by insufflation into the mesenteric arteries. The air passed by the portal vein into the liver, and immediately appeared in minute bubbles in the interlobular spaces, giving a pale, mottled appearance to the surface of the organ.

The animal was allowed to remain alive for a few minutes, and during this time the appearance of air in the interlobular spaces disappeared altogether. The animal was then killed, and air was found in the veins of the liver, and even in the right side of the heart and the pulmonary artery.

In another instance, air was blown very forcibly into the mesenteric veins of a cat, and appeared, after about five minutes, in the right cavities of the heart.

In a third case, however, though the air was thrown with some force into the mesenteric artery, it did not appear afterward, either in the interlobular spaces of the liver, or in the right cavities of the heart.

It is the capillary vessels, therefore, that create an obstacle to the passage of air in the blood. Usually this obstacle is complete; but sometimes, if the pressure be unusually great, or if the animal survives a certain time, a little of the air may pass through the lungs, and appear in the left side of the heart.

Now let us see, gentlemen, how it is that air gains access to the veins in surgical operations. There are two modes in which this accident may occur. The first is by the careless use of the syringe in making injections of medicinal substances into the veins. You are aware that this method of administering remedies has been suggested, and occasionally practiced, in certain morbid conditions. The transfusion of blood has also been resorted to in cases of extreme exhaustion from hæmorrhage. In cholera, saline solutions have been injected into the veins, for the purpose of restoring to the blood the fluids which it has lost by diarrhœa.

In these cases, if the ordinary syringe be used to make the injection, a little air is very apt to be drawn into it, together with the liquid solution, when the syringe is filled; and unless particular care be taken, some of this air will be thrown into the veins, either at the beginning or at the end of the injection. In order to avoid this danger, the syringe, before introducing it into the vein, should always be held for a moment in a vertical position, with the nozzle pointing upward. The air which it contains will thus float upward toward the opening of the tube, and by gently pressing the piston all the air can be driven out in advance of the liquid. The nozzle of the syringe should then be introduced into the vein, taking care meanwhile to maintain a continuous pressure upon the piston, so as to keep the point of the instrument filled with liquid. When once the nozzle is fairly secured in the vein, the injection may then be made without fear.

But there is another mode in which air finds its way into the blood in surgical operations, which cannot be so easily guarded against; that is the accidental or *spontaneous* introduction of air into the veins. In these cases the air enters, of itself, into veins which are wounded in the ordinary way, in the course of surgical operations. Let us see how this accident happens, and what precautions are required to guard against it.

It has been known for many years that the wounding of veins *in certain parts of the body* is attended with risk of the introduction of air. The first case of this kind, in which the nature of the accident was recognized, happened in the year 1818, at the Hospital St. Antoine, in Paris, under the care of M. Beauchêne. A young man entered the hospital for a tumor, situated at the top of the right shoulder, and extending from the spine of the scapula to the superior border of the second rib. The tumor involved a portion of the clavicle; and in the operation for its removal, as this bone was found to be diseased, it was determined that a part of it should be exsected. The clavicle was accordingly sawn through, near its articulation with the sternum. It was then lifted out of its bed, and everted, and the dissection continued beneath it, for the purpose of detaching it from the neighboring parts. At this moment a hissing sound was heard, like that of air penetrating into the pleural cavity. The patient, who had hitherto borne the operation remarkably well, immediately grew pale and faint; the pulse became rapid, hard, and irregular, and there were convulsive movements of the limbs. The hissing sound was again heard, during some manipulations which were employed about the wound, and notwithstanding the use of every means for resuscitation, the patient was dead at the end of fifteen minutes.

At the autopsy, it was found that a portion of the external jugular vein had been cut away, just above its junction with the right subclavian. Through this opening the air had introduced itself into the circulatory system, and had destroyed life in the manner which I have already described.

Since that time, other similar instances have occurred in the practice of Dupuytren, Delpech, Castara, Roux, Bouley, and others. Dr. John C. Warren had a case, which he regarded of the same kind, in 1830, and another in 1831; and at the present day, the possibility of such an occurrence is acknowledged by all practiced surgeons. In the case of Delpech, the body was examined next day, after having been submerged in a large vessel of water. The right side of the heart, which was very much distended, was opened beneath the surface of the water, and the gaseous bubbles which escaped were collected in bell-glasses. They were afterward examined, and found to consist of atmospheric air.

In the case of M. Roux, the gases from the right ventricle were collected, and examined in a similar way, and were also found to consist of atmospheric air.

Now, there are various facts in relation to this accident, which it is necessary to bear in mind. In the first place, spontaneous introduction of air into the veins will not take place, as a general thing, except *in the immediate neighborhood of the chest.* In all the cases which I have cited, the operations in which the veins were wounded were done upon the neck, the shoulder, or the axilla. Most of them were for the removal of tumors in these situations. The introduction of air into a wounded vein, therefore, is liable to take place just in those situations *where the veins are emptied by the movements of inspira-*

*tion.* At a distance from the chest, this accident does not happen, because at that point there is no violent suction force exerted upon the vessel; and if there were, the walls of the vein would collapse beyond the wound, rather than allow the air to pass into its cavity. But if a vein be opened in the axilla, at the upper part of the chest, or in the lower part of the neck, then, at the movement of inspiration, when the blood is violently drawn toward the chest, the air is also drawn into the opening of the vein.

Amussat, who has written an extremely valuable memoir on this subject, insists particularly upon the fact that the introduction of air is liable to take place only in the region which I have mentioned; and he calls it, therefore, the "dangerous region." For, while the veins may be wounded with impunity elsewhere, the division in that neighborhood is always attended with serious risk.

But there are various circumstances which increase this risk, even in the "dangerous region" of the neck and shoulder. One of these is, anything that tends to *hold the vein open*, and thus to prevent the collapse of its walls. If the lips of the wounded vein be held apart by the blades of a forceps, air is much more likely to enter than if they were allowed to collapse. The history of the surgical accidents of this nature, which have occurred thus far, shows that this cause had a great deal to do with producing the result. In nearly all the recorded instances, the wounded vein was either surrounded by a tumor or adherent to its surface; and during the operation the tumor was forcibly lifted or drawn away from its bed, thus stretching the vein, and preventing its collapse. In Beauchêne's case, the introduction of air happened while the clavicle was being drawn outward, and the fibrous tissues about the vein thus put upon the stretch. This should always be regarded, therefore, as a dangerous proceeding, when veins are liable to be wounded in the operation.

If the parts about the vein be infiltrated with cancerous deposit, or by chronic inflammation, this would also render the accident more liable to happen; and might even extend the limits of the dangerous region to a considerable distance from the chest. Every one has observed, in dissecting a limb affected with chronic inflammation of the joint, for example, that the veins running through the infiltrated and hardened tissues have lost their power of collapse; and that they stand open, like arteries, after being divided. This is the condition known by the French as *canalization* of the veins. If such a condition of the parts were to extend upward from the limb, as far as the chest, the influence of inspiration on the veins might be felt throughout the entire intervening distance.

We must remember, furthermore, that air is drawn into the veins only by a forced and violent inspiration. No doubt, the accident is more likely to happen during an operation done without ether, than where a patient is under the influence of an anæsthetic; for while the respiration is calm and equable, the suction force exerted upon the veins, as we have seen, is easily compensated by the flow of blood from behind. But when a man is suffering pain, he is apt to take quick, forced, and sudden inspirations, and it is in such inspirations that air is most likely to be drawn into the vein, and thus become mingled with the blood.

Another circumstance that increases the danger of a fatal result, is an exhausted condition of the patient. From what I have already said, you will understand that the introduction of a small quantity of air into the veins does not necessarily produce death. It may be disposed of by solution, either at once or gradually. The threatening symptoms first produced may pass off, and the circulation return to its ordinary condition. I have often been surprised to see how much air may be blown into the veins of dogs or cats, without immediately causing death.

This has led to the belief, in some instances, that a small quantity of air in the veins was much more liable to be fatal in man, than in the lower animals. But it is the opinion of Amussat that the accident is much more apt to prove fatal when the patient is already exhausted by disease, or by the pain of a long operation. Then, a slight obstruction of the pulmonary circulation will be sufficient to cause death, while it might otherwise have been overcome, after a time, by the force of the heart's action.

At all events, there have been cases of recovery after the spontaneous introduction of air, even in the human subject. In one of Dr. Warren's patients, the most alarming symptoms continued for thirty minutes; but the patient nevertheless recovered, and the operation was afterward successfully finished. Other instances of recovery have occurred in the hands of Clémot, of Delaporte, of Amussat, of Dr. Mussey, and Dr. Mott. In a case which happened to Malgaigne, the patient recovered from the immediate effects of the accident, but died four days afterward, by an accumulation of frothy mucus in the bronchial tubes.

Now, in regard to the *symptoms* which accompany this accident, there is a great similarity between the different cases. At the moment when the air finds entrance into the vein, a peculiar noise is heard, like the passage of the wind through a small opening, or of airbubbles through a liquid. It is spoken of, by different observers, as a "hissing," "whistling," "lapping," or "gurgling" noise. In several instances, it has been compared to the sound of air entering a partially exhausted receiver. It has been mistaken, once or twice, for the passage of air into the pleural cavity; and in Beauchêne's case it was supposed, until after the autopsy, that the pleura had actually been opened. Sometimes the sound occurs but once; sometimes it is repeated at several short intervals, as the air is drawn in by successive inspirations.

At the same time with the occurrence of this sound, bubbles of air are often *seen* mixed with the blood in the wound, or regurgitating from the vein at the moment of expiration. In the case of M. Ulrich, frothy blood was discharged from the opening in the vein. In one of Dr. Warren's cases, bubbles of air appeared in the blood at the wound. M. Rigaud himself saw the air enter the vein and displace the liquid; and in Dr. Mussey's case, air-bubbles were also observed to pass into the vein. Most frequently, however, the air gains entrance too suddenly to be actually seen by the observer, and the hissing or gurgling sound is the only thing that attracts his notice.

It is curious, that the patient himself is almost always conscious that some terrible and threatening accident has occurred. He sometimes even feels that the trouble is located in the heart or in the vessels. In Beauchêne's case, the patient made a singularly striking exclamation. He cried out, as soon as the air had penetrated the vein, "*My blood is falling into my heart! I am a dead man.*" (Mon sang tombe dans mon cœur! Je suis mort.) In one of Dr. Warren's cases, the patient called out suddenly, "*I am sick!*" Delaporte's patient exclaimed, "*Ah! my windpipe is cut! I am lost!*" In the case of M. Amussat, the patient, who was a woman, survived the accident. She was afterward interrogated by the surgeon, as to whether the difficulty appeared to her like a fainting-fit. She replied "No;" that "in a fainting-fit, the trouble comes from above; but in that, the trouble came from below."

Very often, instead of saying anything, the patient utters a cry of distress, and becomes pale, insensible, and convulsed. There is frequently a similar cry, with convulsions, when air is blown into the veins of the lower animals.

It is unnecessary for me to say, gentlemen, that when air is introduced spontaneously into the veins, in this way, in surgical operations, it destroys life in precisely the same manner as when it is blown in artificially, for purposes of experiment. It passes to the right cavities of the heart, blocks up the vessels of the lungs, and stops the pulmonary circulation. It is accordingly found in the right auricle and ventricle, after death, and is seen also in greater or less abundance in the neighboring veins.

Furthermore, this accident may be followed by recovery, in the human subject, as well as in the lower animals. Amussat has collected, in his monograph, twelve cases in which death was not produced. In three of these instances, though the sound of air entering the vein was perfectly distinct, no injurious result followed, and the patient did not even know that he was in danger. In the other nine cases, however, the patient suffered for a time very alarming symptoms, which afterward passed off. No doubt the fatal result depends, in the human subject also, on the quantity of air introduced, and the exhausted condition of the patient.

It is plain, therefore, what ought to be done when air happens to have been introduced into a vein in a surgical operation. As soon as the sound is heard which indicates the passage of air, the opening in the vein should be at once closed by the finger of the operator, and the vessel secured by a ligature as soon as possible. For the danger does not cease with the first ingress of air; at any succeeding inspiration a new quantity may be drawn into the vein; and we have already seen that the risk of a fatal result is in direct proportion to the amount of air which gains access to the vascular system.

Attempts have occasionally been made to extract the air from the veins, after its admission, by introducing a syringe and withdrawing the piston; but the success attending these efforts has not been sufficient to encourage their repetition.

A few words, gentlemen, with regard to the *post-mortem appearances* in fatal cases of this sort, and the means of distinguishing them from others of a different nature. It is sometimes a matter of doubt whether a patient has perished from the introduction of air into the veins, or from some other cause. Now, how are we to determine this point?

In the first place, it is important to know that air may be introduced into the veins *after death*, and during the progress of the autopsy. You will hardly ever make a post-mortem examination of the head, for example, without finding air-bubbles in the superficial veins of the cerebral hemispheres. After sawing through the skull, you lift the calvaria forcibly upward, cutting or tearing away the attachments of the dura mater and the longitudinal sinus. It is at this moment that air is introduced into the longitudinal sinus and the veins which open into it. It can afterward be seen in the vessels ramifying over the surface of the cerebral hemispheres.

The same thing often happens when you open the chest. While raising the sternum, you draw the air into the superior vena cava and the subclavian veins, through any opening which may have been made in them; and when the chest is fairly opened, you may see the air-bubbles, sometimes in considerable quantity, in the large veins in its interior. The mechanism of the introduction of the air, in both these cases, is the same as when it happens during life, viz., by the forcible lifting of a bony frame-work or inclosure, by which the air is sucked or drawn in from the exterior. It may be drawn into the abdominal veins, also, in the same way, if the manipulations used are such as to produce a similar effect.

How are we to know, therefore, when we find air in the veins in the interior of the body, whether it has been introduced during life, or accidentally gained admission after death?

You can determine this point by observing the physical condition of the air and of the blood. When air is drawn into the veins during an autopsy, it is present merely in the form of large bubbles—mostly in the superior and inferior vena cava, in the neighborhood of the heart. Even if drawn into the cavity of the heart itself, it presents the same appearance, having been mechanically introduced after the vital actions had ceased. But when it is introduced during life, while the circulation is going on, it is carried, as I have already told you, directly to the right cavities of the heart; and there, by the movements of the organ, it is whipped up into a bloody froth, or foam, consisting of minute air-bubbles, intimately mixed and entangled with the blood. *It is the presence of this bloody foam in the right cavities of the heart* which shows that the introduction of the air has taken place during life. This ultimate mixture of the air with the blood is caused only by the movements of the living organ; and there is no other appearance that can be considered as conclusive in regard to the nature of the accident.

There is one other cause, however, which may give rise to the appearance of air-bubbles in the blood after death. That is, the process of putrefaction. If the autopsy be delayed for some days after death, if the weather be warm and the body exposed, putrefactive gases may be developed in the blood, and may show themselves in the form of air-bubbles. But in such a case, the odor of decomposition in the body, as well as other marks of putrefactive change, would be sufficient to indicate the real nature of the appearances. To prevent all mistake, however, it would be well, in doubtful cases, to collect the gases found in the blood and to analyze them, as was done by Roux and Delpech, in the instances which I have already mentioned.

A very ingenious rule is given by Amussat for distinguishing the gases produced by putrefaction, from air which has been introduced into the heart during life. When the heart is distended, he says, with air introduced during life, the cadaveric rigidity of the cardiac fibres comes on afterward; and then, even if you allow the confined air to escape, the organ still preserves its expanded form. But the distention of the heart by putrefactive gases is accompanied with a relaxed condition of its fibres, and on puncturing it, the organ immediately collapses, owing to the flaccid condition of its muscular parietes.

In conclusion, gentlemen, I have to mention to you one very curious way in which the introduction of air into the vessels has been thought to take place; that is, *by the veins of the uterus after parturition.*

In Amussat's monograph on this subject, a number of cases are mentioned, in which sudden death occurred in young females, after delivery, without any apparent cause; and the author expresses his conviction that, in some of them at least, the death was owing to the introduction of air into the uterine veins. A similar instance is quoted by Milne Edwards, in his book on Physiology, from Lionet. Dr. J. R. Cormack wrote a paper on the same subject, in 1850, which was published in the Edinburgh *Monthly Journal of Medical Science*, for that year. Dr. Cormack maintains the possibility of air entering the uterine veins after delivery, and quotes cases from Dr. Meigs, Dr. Lever, and Dr. Simpson, to show that it has occasionally happened. Dr. Simpson explains it by supposing that the uterus itself, by alternately relaxing and contracting, *forces* the air into the open mouths of its own vessels.

But, on looking over the whole history of this subject, I must acknowledge that it appears exceedingly doubtful whether such an accident ever actually happened in the human subject. I am not aware that there is a single case on record in which the evidence was conclusive. In most of the cases there was, at the same time, more or less hæmorrhage, to which the death might be attributed. In many of them there was no post-mortem examination. In some, air was found in the veins of the uterus and abdomen; but in none of them was the frothy mixture of blood and air found in the right cavities of the heart, which can alone show conclusively that the introduction of the air took place during life. On the whole, therefore, it is very uncertain whether such a spontaneous introduction of air, after delivery, has ever been the cause of death in the human female.

A very remarkable case, however, occurred at Albany, in this State, a few months ago, in which it seems certain that air was really introduced into the uterine veins, though by a very different mechanism from that suggested by Dr. Simpson. The case happened under the observation of Dr. John Swinburne, who published an account of it in the Philadelphia *Medical and Surgical Reporter*, for April 23d, 1859. The patient in this instance was an unmarried woman, who was residing at the house of an abortionist, (also a female,) for the purpose of having a miscarriage induced. An attempt was made to rupture the membranes, by introducing into the uterus a gutta-percha catheter; and, according to the account given by the woman, no sooner was it introduced into the uterus, than the patient fell back, as she supposed, in a fainting-fit. Finding that the fainting-fit did not pass off, the woman became alarmed and sent for a doctor, who immediately repaired to the house and found that the patient was dead.

Dr. Swinburne, next day, made an examination of the body, and found that the sinuses of the uterus, the jugular veins, and even the superficial veins of the body, contained air. The right cavities of the heart were "distended with a spumous mixture of blood and air." The uterus contained a healthy, five-months fœtus. The membranes were unbroken, but were separated from the uterine wall externally, on the right side, together with a portion of the placenta; and there was a perforation at this spot, leading into the uterine sinuses, about two inches distant from the cervix.

The air found in these vessels could not have been the result of decomposition, since the affair happened in the month of March, and the autopsy was made fourteen hours after death.

Dr. S. expresses the opinion, and, I think, correctly, that in this case the air was forcibly *blown in* through the catheter, with the intention of producing separation of the membranes. The membranes being partially separated in this way, and the uterine veins passing to the placenta, being opened, but not quite torn across, air might readily be forced into the veins by insufflation, and might so produce instantaneous death. Or, the end of the instrument may have penetrated one of the uterine sinuses, and thus may have introduced the air directly into the vascular system.

